# Proprietary Medicines

**Published:** 1882-10

**Authors:** 


### PROPRIETARY MEDICINES.

It is plain enough that no good physician in regular standing would belittle his high profession by becoming a proprietor of any medicament, but what can we say when it comes to recommending such? Upon taking up any medical magazine we are confronted by the names of prominent gentlemen of the profession appended to patent medicines. That the sale of these drugs is a growing evil and one which has assumed gigantic proportions is self-evident, and the feeling is growing among the profession that some step must be taken to limit it. It is also evident that as yet we have not reached any satisfactory means of procedure.—*Chicago Medical Review.*

